# Cash assistance programming and changes over time in ability to meet basic needs, food insecurity and depressive symptoms in Raqqa Governorate, Syria: Evidence from a mixed methods, pre-posttest

**DOI:** 10.1371/journal.pone.0232588

**Published:** 2020-05-07

**Authors:** Kathryn L. Falb, Alexandra H. Blackwell, Julianne Stennes, Jeannie Annan

**Affiliations:** International Rescue Committee, Washington, DC, United States of America; Washington University in St. Louis, UNITED STATES

## Abstract

Raqqa Governorate has been grappling with dual crisis-related burdens from the civil conflict and ISIS occupation. As part of a response to support households within this area, a three-month, unconditional cash assistance program was implemented by the International Rescue Committee to help households meet their basic needs. A quantitative, pre-posttest with 512 women at baseline (n = 456 at endline) was conducted in northern Raqqa Governorate between March-August 2018 to determine their experiences in this cash assistance program and to understand perceived change over time in food insecurity, perceived household serious needs and daily stressors, and depressive symptoms before and after cash was delivered. Forty women also completed in-depth interviews using a life line history technique at endline. Linear household fixed effects models demonstrated significant reductions in food insecurity (β = -0.95; 95%CI: -1.19–-0.71), no change in perceived serious household needs and daily stressors (β = 0.12; 95%CI: -0.24–0.48), and increases in depressive symptoms (β = 0.89; 95%CI: 0.34–1.43) before and after the period of cash distribution. Although no causality can be inferred, short-term emergency cash assistance programming yielded significant improvements in food security, was highly acceptable and viewed favorably, and assisted women and their families to meet their basic needs in this emergency setting. However, before and after this form of cash assistance was implemented, no meaningful changes in the perceived levels of serious needs and stressors amongst households were observed, but potential increases in depressive symptoms for women were reported during this time period. Further work is needed to determine appropriate targeting, length, and dosage of cash, alongside any potential livelihood, psychosocial, or structural complementary programming to yield potential positive mental health benefits of a cash assistance program focused on meeting a population’s basic needs while not inadvertently delaying or decreasing reach of life-saving cash assistance programming in emergencies.

## Introduction

Since the outbreak of civil conflict against the Government of Syria in 2011, and the subsequent rise of the Islamic State of Iraq and Syria (ISIS), Syria’s population has been exposed to waves of violence, widespread displacement, and economic disruption. More than 6 million Syrians have been internally displaced and another 7 million have fled to other countries. At least half a million people have died in the conflict. [1]

The region of Raqqa Governorate was particularly hard-hit by the conflict, enduring a years-long occupation by ISIS which began in 2013 and ended in October 2017, when a US-led coalition and Kurdish forces finally succeeded in liberating the city of Raqqa from ISIS control. As the campaign to expel ISIS from Raqqa City escalated from late 2016 through late 2017, nearly all of the city’s 375,000 residents were displaced as most of the city was destroyed by the fighting. Residents of Raqqa City fled to other towns and villages within the Governorate, areas which had been liberated from ISIS in the months preceding the battle for Raqqa City. [2] As of August 2018, most of those who fled from Raqqa City continued to live in displacement, while about 150,000 had returned to Raqqa City, despite continued danger from unexploded mines and a general lack of economic opportunity or humanitarian support available in the city. [1] While data is scarce, programmatic reports and investigations reveal that women and girls faced increased violence against them due to diminishing gender equitable norms and freedoms. [3]

Across Syria, the conflict has had a destructive effect on the economy. After recording 3.4% GDP growth in 2010 (the last year before the start of the civil conflict), Syria experienced an estimated 63% total decline in GDP between 2011 and 2016. [4] This steep decline in GDP was caused by a confluence of war-related factors including external implementation of sanctions against Syria, destruction of infrastructure, decline in oil production, lack of business confidence, and displacement of skilled and unskilled workforce. [4] Communities reported that lack of income is a major challenge, and households were left struggling to cover costs of housing, utilities, fuel, water, and food. [5] Household vulnerability assessments conducted by the International Rescue Committee (IRC) in Northeast Syria (NES) revealed that the conflict and related destruction significantly disrupted household livelihoods in the region. Internal program data show that, though most women were not in paid work before the war, increased insecurity during and after the war and ISIS occupation led to an even smaller minority of women earning an income post-conflict, alongside a loss of livelihoods for men due to the war.

Along with a general contraction in the economy and loss of cash income, agricultural productivity and food security had also been damaged by the conflict. The agriculture economy shrunk by approximately 40% between 2011 and 2015. [4] Wheat production was 55% lower than the pre-conflict average and according to the Food and Agriculture Organization and World Food Programme, there were “30% fewer cattle, 40% fewer sheep and goats, and 60% less poultry” than the livestock populations that existed before the start of the war. [4] Coupled with loss of cash incomes for many households, this drop in agricultural production contributes to widespread food insecurity across Syria.

This economic instability, as well as prolonged conflict experiences including displacement and violence, have resulted in poor mental health among Syrian refugee populations. [6–8] Recent studies show high prevalence of depressive symptoms (from 37–44%) and post-traumatic stress symptoms (from 33.5–38%) among Syrian refugee populations. [9–11] The protracted conflict setting has continued to impact the health of Syrians; a recent literature review of mental health issues amongst Syrians affected by armed conflicted demonstrates that challenges adapting to the post-emergency context may have increased their risk of poor mental health. [12] Existing evidence is primarily from Syrian refugee populations residing outside of Syria; few studies have systematically measured the mental health symptoms of populations residing inside the country since the start of the conflict.

### Cash assistance programming

Research has shown that cash assistance programming can help conflict-affected households withstand economic shocks, unemployment, and fluctuating markets associated with conflict and recovery. [13] Households receiving cash transfers are less likely to cut back on food consumption or engage in negative coping such as selling assets and taking children out of school to work. [14,15] Evidence also suggests that cash transfers have a consistent positive effect on food security, and in crisis settings with functioning markets, cash transfers can be more effective than in-kind food distribution at improving food security outcomes. [14]

In stable development settings, cash programming can improve beneficiaries’ mental health by reducing their level of financial stress by helping them meet their basic needs. A study of the impact of cash transfers in Kenya found that receiving cash produced significant increases in reported happiness and life satisfaction, and reductions in reported stress and depression through self-reported survey measures, although cortisol levels did not demonstrate an overall effect. [16] Evidence from Malawi also reveals a beneficial relationship between cash transfers and mental health. Beneficiaries of a national program providing unconditional cash transfers to the poorest households saw a large and significant reduction in depression among youth in households receiving cash, with the largest effect observed among young women. [17] A review of studies examining the influence of cash transfers on IPV found that in development settings, cash assistance has the potential to decrease IPV across populations through multiple pathways, including improving the bargaining power of women and girls by transferring cash directly to them, or by reducing economic stressors within the household. [18] However, there is a dearth of evidence on programs from humanitarian and conflict settings, where cash programs are generally shorter and less targeted than cash programs in development settings.

### Objective

Given gaps in knowledge of the impact of cash assistance programs on the economic and mental health conditions of women living in an acute conflict setting, this study sought to determine the perceived impact of cash transfer programming in Raqqa Governorate, Syria on the ability of households to meet basic needs, food insecurity, perceived serious household needs and daily stressors, and depressive symptoms amongst women.

## Methods

### Study design & sample

The present study was conducted between March–August 2018 in Raqqa Governorate, Syria as part of the What Works to Prevent Violence Against Women and Girls research program. Data were gathered using a pre-posttest design, in which women from cash programming beneficiary households were interviewed before and after their households received three monthly unconditional cash transfers. As the study was intentionally conducted in a setting that mirrored an acute humanitarian emergency, protocol shells were developed in advance of implementation in order to not delay potential life-saving programming. Protocols were designed for a randomized control trial, a regression discontinuity, and a pre-posttest approach that could have been implemented based on particulars of different humanitarian emergency settings. During this pre-positioning period, locations for a study were also being scoped in which cash assistance programming was being delivered and IRC women’s protection and empowerment programming were also present to ensure ethical conditions could be met. While Northeast Syria met these criteria, based on assessments of logistical and ethical constraints, designs allowing for more causal inference were not feasible nor ethical and, thus, a mixed methods pre-posttest design was selected for this study. Further details regarding the selection of the study design are found in the full report. [19]

The study sample comprised of all households that had a woman aged 18–59 years from a beneficiary household. Female heads of household between ages 18–59 were selected to be interviewed as a representative of their household when possible. In male-headed households a Kish grid was used to select the female respondent in cases where more than one eligible woman aged 18–59 was residing in the household. Respondents were interviewed in March or April 2018 prior to the first cash payment and again in August 2018, approximately two-three weeks after the third and final payment.

Qualitative interviews using a life line history methodology were conducted alongside the quantitative interviews at endline. Maximum variation sampling was used to select women to participate in the qualitative interview. The aim of the maximum variation methodology was to capture a diverse set of experiences of violence, displacement status, age, and female- vs male-headed households. Further information about the qualitative sample can be found in the S1 Table.

### Intervention

The cash transfer amount, set by the regional cash working group, was a monthly transfer of $76 for three months, which was intended to cover approximately 80% of non-food items a six-person household may need. Households were selected by IRC’s Economic Recovery and Development team to participate in the cash assistance program based on pre-defined economic vulnerability criteria. Vulnerability criteria considered for this program included indicators such as income, asset ownership, food insecurity, and receipt of remittances from abroad. Transfers were given to the head of household; the program did not dictate whether it had to be a female or male head.

### Data collection

The IRC hired external female data collectors as part of the quantitative study. IRC research staff trained enumerators on survey methodology, consenting practices, and other research related activities. The IRC Women’s Protection and Empowerment team trained the data collectors on gender-based violence principles and referral pathways. The questionnaire was administered by these enumerators asking each question aloud and recording the answers using the data collection application SurveyCTO. All survey items were translated and backtranslated into Arabic from English. All interviews took place in a private setting after the completion of informed consent.

The qualitative interviews were conducted with 40 women in a private room by female IRC research staff or gender-based violence caseworkers not involved in the cash program. IRC staff led this data collection effort for qualitative interviews due to the sensitivity of the topics discussed. Interviews were conducted with one interviewer and one note-taker, with the exception of those interviews where an Arabic translator was present when led by English-speaking only IRC research staff (6 of 40 interviews). Only written notes were taken due to non-acceptability of audio recording devices. Notes and visual history diagrams were translated from Arabic into English.

All study tools and protocols were approved by the International Rescue Committee’s Institutional Review Board (WPE 1.00.002) and by local access authorities. Care was taken to describe that their decision to participate would have no influence on current or future assistance from the IRC.

### Measures

#### Quantitative

Food insecurity was measured using the Household Food Insecurity Access Scale (HFIAS) [20]; reliability of the scale has been documented in other humanitarian settings [21–23] The HFIAS tool includes nine binary items, asking the respondent about the quantity and quality of the food consumed in their household in the past four weeks (For example: In the past four weeks, did you worry that your household would not have enough food? Or: In the past four weeks, did you or any household member go to sleep at night hungry because there was not enough food?). Responses were summed to form a continuous measure 0–9. The scale had a Cronbach’s alpha of 0.83 in the current study.

Perceived serious household needs and daily stressors was assessed using an adapted Humanitarian Emergency Settings Perceived Needs (HESPER) Scale. [24] In the present study’s adapted version, this scale involves asking the respondent whether they feel they have a “serious problem” regarding 20 different items relevant to basic needs in a humanitarian setting, including food, clothes and shelter, as well as physical health, safety, education and social issues. (For example: Do you have a serious problem because you do not have a suitable place to live? Or: Do you have a serious problem with your physical health, for example, because you have a physical illness, injury or disability?) Each of the 20 items were assessed in a binary manner and summed in a final, continuous measure to assess perceived serious household needs and daily stressors. The original scale has been validated in a number of humanitarian settings, including Jordan, Nepal, and Haiti. [25,26] In this region in northeast Syria, the HESPER scale had an acceptable Cronbach’s alpha of 0.71.

Depressive symptom score was calculated using the nine-item Patient Health Questionnaire (PHQ-9). [27] Each item is given a value of 0 to 3 reflecting how often the respondent has experienced each depression symptom in the past two weeks: not at all (0), several days (1), more than half the days (2), or nearly every day (3). (For example: Little interest or pleasure in doing things; or: feeling down, depressed or hopeless). All items are totaled to create a single score ranging from 0 to 27, with higher scores indicating worse depressive symptoms. Validated in other humanitarian settings [28–30], this scale has demonstrated a Cronbach’s alpha of 0.73 in the present analysis.

Continuous demographic measures include age in years and number of children, as well as binary measures of ever attended school, currently displaced, and female headed household status. Categorical baseline demographics included marital status and women’s livelihoods. To assess disability status, an adapted version of the Washington Group Disability Short Set was used, which identifies individuals with a greater risk for participation restrictions than the general population. [31] The measure labels a person as having a disability if they report having some or a lot of difficulty doing any of the core functions or cannot do them at all.

#### Qualitative

Qualitative in-depth interviews were conducted at endline to obtain a deeper understanding of the experiences of women during the cash assistance program, particularly in relation to ability to meet basic needs and spending habits, and to further explore their experiences of violence. The qualitative interview guides were open-ended and a visual timeline technique was used to ask women about their lives and experiences before, during and after the conflict, and before, during and after the cash assistance program.

### Analysis

Descriptive statistics were generated for demographics at baseline and outcome variables (individual items of outcome variables and frequencies are found in S2–S4 Tables). Less than 0.3% of all observations were missing within outcome scales. For all continuous outcome measures, missing responses to individual items were coded as 0 for conservative estimates. Potential changes between baseline and endline in outcomes of interest were assessed in generalized linear models which account for clustering at the individual level. The first model presents unadjusted coefficients of change over time, the second model presents adjusted coefficients of the time variable after controlling for women’s age, ever attended school, currently displaced, female headed household status, moderate or severe disability status, and marital status; the adjusted model employed a complete case analysis approach where participants with missing demographic data were dropped from the models. The final model presents coefficients adjusted for household fixed effects to account for both measured and unobserved non-time varying variables.

Qualitative data were analyzed using a combination of a priori and inductive coding of the transcripts and interactive timelines drawn by the interviewer. An initial coding structure was developed using the project’s theory of change, and additional codes were developed through grounded theory as patterns emerged from the data. Quotes are used throughout the article to illustrate prominent patterns in the analysis.

## Results

**Fig 1:**
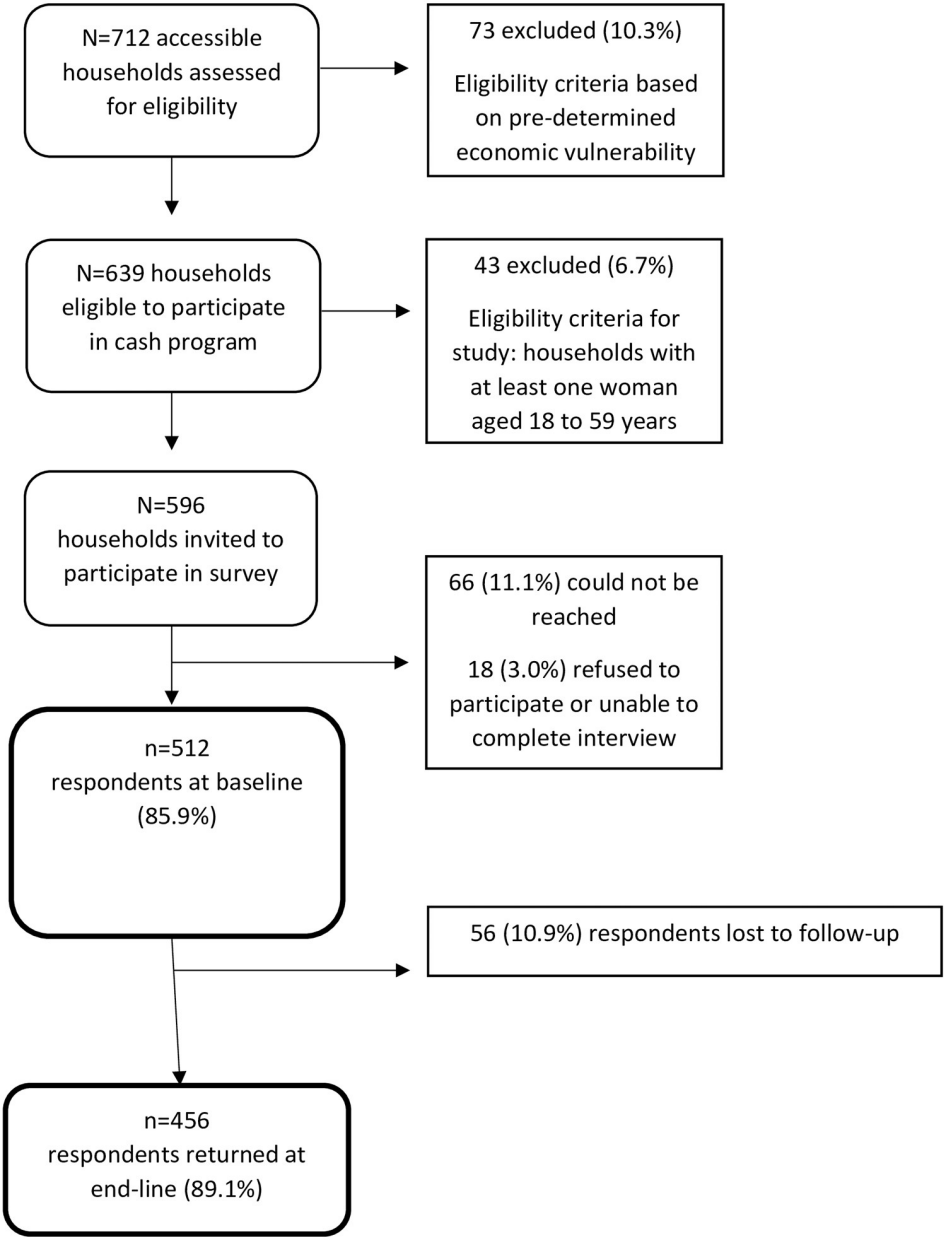
Study sample flow chart.

Among the 596 households deemed eligible to participate in the interview, a total of 512 women were interviewed at baseline, resulting in a 85.9% response rate. After the final cash transfer, 456 respondents (89.1% of baseline sample) returned to complete the endline interview (see Fig 1). There were no significant attrition differences between time points based on baseline demographic sample composition (Table 1).

**Table 1:** Baseline sample characteristics of women at baseline (N = 512).

	Baseline		Endline		Comparison
Demographic Characteristic	N^1^	% (N) / Mean (SD)	N^2^	% (N) / Mean (SD)	P-Value
Age	486	36.03 (10.08)	433	35.75 (10.12)	0.67
Ever Attended School	512	53.91% (276)	456	53.73% (245)	0.96
Has Moderate to Severe Disability	512	30.08% (154)	456	29.82% (136)	0.93
Currently Displaced	511	38.52% (180)	456	33.8% (154)	0.64
Female Headed Household	511	63.41% (324)	456	65.1% (297)	0.58
Marital Status	512		456		0.98
Married		41.80% (214)		40.35% (184)	
Widowed		36.52% (187)		37.06% (169)	
Divorced		12.50% (64)		12.50% (57)	
Separated		3.13% (16)		3.29% (15)	
Single		6.05% (31)		6.80% (31)	
Number of Children	502	3.72 (2.76)	448	3.60 (2.76)	0.51
Women’s Livelihood	511		456		0.77
Housewife/Unemployed		65.95% (337)		64.04% (292)	
Daily Laborer		18.0% (92)		19.74% (90)	
Other		16.05% (82)		16.23% (74)	

^1^ Data may not equal 512 due to missing data

^2^ Data may not equal 456 due to missing data

### 1. Basic needs spending habits

**Fig 2:**
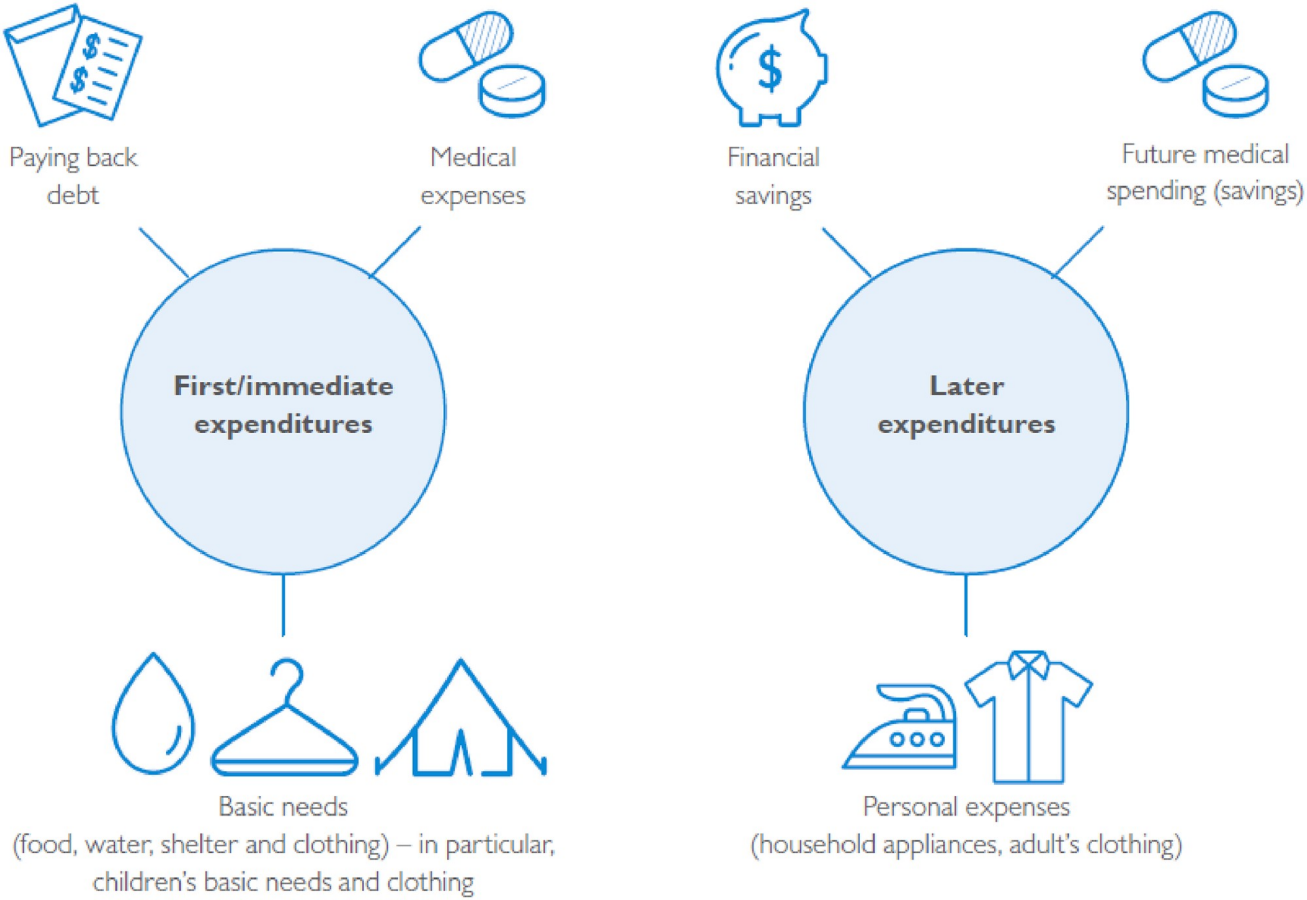
Common expenditures by cash transfer period as described in qualitative interviews.

In qualitative interviews, women reported that their households primarily spent the cash on basic needs (including food and water, shelter and clothing), paying back debts and paying for medical expenses. When analyzed temporally, women’s reported spending priorities changed depending on the order of the cash transfer, as illustrated in Fig 2. [19] The majority of women interviewed reported spending the first transfer, which occurred before the Eid al-Fitr holiday at the end of Ramadan, on basic needs for children and repaying debt. When describing her household spending after the first round of cash transfers, one married woman said, “The money finished in one day, I paid back debts, and bought medication for my husband and children.” During the second and third rounds, which occurred just before Eid al-Adha, while several women reported still spending some portion of their cash on basic needs and debt, more also reported spending on personal expenses for themselves and saving for anticipated medical expenses and future household financial needs. A widowed woman said of her third round of cash transfers: “I didn’t spend it yet, I kept it for emergencies.”

### 2. Changes in past month food insecurity

At baseline, the mean number of items a woman endorsed on the food insecurity scale was 6.76 (SD: 2.21); at endline, the mean number was 5.84 (SD: 2.43) (Table 2). In the unadjusted linear model, between baseline and endline food insecurity items decreased by 0.92 points (95%CI: -1.17–-0.68; p<0.0001). The significant decrease in food insecurity over time remained robust when adjusting for demographics (β = -0.90; 95%CI: -1.14–-0.65; p<0.0001) and when accounting for household fixed effects (β = -0.95; 95%CI: -1.19- -0.71; p<0.0001).

**Table 2:** Changes in food insecurity, perceived household serious needs and daily stressors, and depressive symptoms at baseline and endline over the period of cash assistance programming.

	Pretest Overall	Posttest Overall	Unadjusted Model^1^			Adjusted Model^2^			Household Fixed Effects Model		
	Mean (SD)	Mean (SD)	β	95%CI	P-Value	β	95%CI	P-Value	β	95%CI	P-Value
Food Insecurity (Past Week)	6.76 (2.21)	5.84 (2.43)	-0.92	-1.17–-0.68	<0.0001	-0.90	-1.14–-0.65	<0.0001	-0.95	-1.19 –-0.71	<0.0001
Perceived Serious Household Needs and Daily Stressors	12.08 (3.32)	12.11 (3.87)	0.04	-0.31–0.40	0.83	0.05	-0.33–0.42	0.81	0.12	-0.24–0.48	0.52
Depressive Symptoms (Past Month)	11.08 (5.02)	11.93 (5.08)	0.86	0.32–1.40	0.002	0.92	0.35–1.49	0.001	0.89	0.34–1.43	0.001

^1^ Unadjusted models N = 968; adjusted models N = 899 due to missing values for covariates; household fixed effect model N = 968

^2^ Adjusted for baseline characteristics of female headed household, displacement status, marital status, age, ever attended school, has moderate or severe disability, livelihood, number of children; all models presented adjusted for individual level clustering

All of the women interviewed qualitatively described the deprived conditions of their households before the start of the cash program. One married woman said of this time, “We lived very hard days, we did not even have water to drink, the weather was so hot, we were hungry and thirsty. All of us, women and men, were looking for any piece of food, bread or tomato.” A widowed woman who was working as a seasonal farmer to provide for her household’s basic needs explained that her income was inadequate for purchasing higher quality foods: “What I get barely covers the price of bread.”

When asked how they spent each cash transfer during qualitative interviews, the majority of women interviewed listed the many food items that they were able to purchase with the cash, including rice, oil and even meats. One divorced woman said, “I was able to prepare the meal I wanted.”

### 3. Changes in household perceived serious needs and daily stressors, and depressive symptoms

On average, women at baseline agreed with 12.08 statements (SD: 3.32) on the 20 item HESPER scale, signifying high household perceived serious needs and daily stressors (Table 2). At endline, this remained virtually unchanged (Mean: 12.11; SD: 3.87). Unadjusted, adjusted, and household fixed effects models were not significant (β = 0.04; p = 0.83; β = 0.05; p = 0.81; β = 0.12; p = 0.52, respectively).

Several women who were interviewed qualitatively reported that, before receiving the cash, they were suffering from stress on a regular basis due to the constant difficulty of meeting their families’ basic needs. After receiving the cash, they stated that they experienced a temporary relief from this stress. One divorced woman expressed, “I felt like I was imprisoned and released [because of the cash]. … I was relaxed.”

Other women reported that the cash was not enough to cover all of their immediate needs, and that they had to continue to borrow money and take out debts even while participating in the cash program. This was reported more among widowed and divorced women than married women in the qualitative interviews. A widowed woman who was describing this challenge said, “The money wasn’t enough because I gave [my daughter] back her money and I borrowed money again to cover the expenses of the month.”

In terms of women’s individual changes in depressive symptoms, on average, women had a mean of 11.08 on the PHQ scale at baseline (Table 2). At endline, an average of 11.93 symptoms were reported. In the unadjusted model, between baseline and endline, depressive symptoms changed by 0.86points (95%CI: 0.32–1.40; p = 0.002). A similar change was noted in the adjusted model (β = 0.92; 95%CI: 0.35–1.49; p = 0.001) and in the household fixed effects model (β = 0.89; 95%CI: 0.34–1.43; p = 0.001).

While the program was not designed to improve mental health, several women who were interviewed qualitatively reported that receiving the cash transfers reduced their stress during the period of cash assistance delivery and relieved them of the feelings of shame and humiliation they had felt when they had to rely on family members or neighbors for financial support, or had to resort to borrowing money or begging. One divorced woman living with her in-laws stated, “The cash we received maintained our dignity and met our needs. I don’t live like a queen because of the aid, but it is good.” Another divorced female head of household described receiving the cash as ‘a mountain off [of] my back’. A few women also described the power they felt from gaining more financial responsibility as a result of receiving the cash transfers.

However, even among those who reported that household needs were met during the cash program, most women who participated in qualitative interviews reported that they felt stressed and anxious about the cash transfers ending. Respondents acknowledged that the economic relief was temporary and reported concerns about having to continue borrowing money, the lack of local employment opportunities, and therefore being unable to provide for their families’ basic needs once the program ended. One married woman who provided the sole income for her household said, “I didn’t think yet [about] what I’m going to do when the project ends. I’ll work in the field from the early morning to the evening, and I’ll get 500 liras. I will suffer and struggle again.”

### Discussion

Households in Raqqa Governorate, Syria overwhelmingly spent cash transfers on basic needs, including food, clothing or related medical and health needs, which is consistent with other literature on unconditional cash transfer expenditures. [32,33] Also aligned with previous research, reductions in food insecurity after receipt of cash were found [34], which in some development schemes have resulted in further reductions in malnutrition and other related outcomes. [35] These spending habits were consistent with the primary intention of short-term emergency cash assistance programming in helping households meet their basic needs. The present study did not capture these nutrition-related outcomes, thus, it remains unclear whether increased food security could improve potential malnutrition outcomes within more fragile settings, as recent studies from Somalia internally displaced persons camps and in Niger found improvements in food security, but no change in malnutrition amongst young children. [36,37] Further longitudinal research is needed to provide nuanced understanding of how cash, including delivering modality and dosage, and contextual factors may influence nutrition related outcomes, beyond food insecurity. [38]

While we are only able to assess changes in depressive symptoms and perceived household needs and daily stressors over time without a comparison group, data point to a potential relationship between cash assistance and mental health outcomes both during and after the assistance. Qualitatively, some women described cash meaningfully reducing the daily stressors facing women and their families. However, quantitative changes in household-level stressors and perceived serious needs remained stagnant and women’s reported individual depressive symptoms showed a small increase. As this short-term emergency cash program was not designed to improve these outcomes, it is not necessarily surprising that it was not of sufficient dosage or length to realize positive improvements in these metrics.

Nonetheless, recent analyses from cash transfer research in Zambia demonstrate similar findings in improvements in food security, but not in perceived stress; authors infer positive changes in other outcomes are not sufficiently changed to impart overall reductions in stress. [39] Future research should determine whether there is a threshold or tipping point to achieve changes in mental wellbeing from cash assistance programming, as other studies have largely shown positive improvements. [40] For instance, a study in Kenya assessed the mental health effects of cash by testing participant levels of the stress hormone cortisol. Results from cortisol testing demonstrated no meaningful change overall,^17^ but sub-analyses revealed additional details of how program design can affect the impact of cash on mental health. Significant reductions in cortisol were observed when the cash was targeted towards the female, disbursed in larger amounts, and given as a monthly payment rather than a lump sum, likely due to increased stability and certainty of funds.^17^ However, survey responses of depressive symptoms found an overall average improvement of symptoms, but no significant differences based on female or male recipient of cash assistance, suggesting mental health outcomes related to cash assistance programming may be sensitive to both program design elements and measurement approaches.

Further, the population inside Syria has faced extreme adversity and pervasive violence [41]–cash assistance programs likely need to be paired with more intensive mental health, psychosocial support services, or larger structural interventions to impart meaningful changes in the lives of women or more holistically address the challenges they may be experiencing. Additionally, other analyses from the present study demonstrate that during the period of cash transfer delivery, some forms of past-three month intimate partner violence amongst a married sub-sample increased, although causality cannot be inferred due to the lack of counterfactual. [19] Nonetheless, such experiences of violence have been shown to be associated with a nearly three-fold increase in risk of depressive symptoms in this context [42], suggesting a potential dynamic relationship between cash assistance, intimate partner violence, and depressive symptoms in Raqqa Governorate, Syria.

Interpretation of findings should be considered in light of limitations. First, there was no control group within this mixed methods study; thus, causality cannot be inferred and other external factors may have led to the perceived reported changes over time. For instance, other humanitarian organizations may have been operating in the area and providing food assistance to the population which led to the increased food security women reported. Alternatively, cash assistance may have also meaningfully reduced levels of perceived household serious needs or daily stressors or women’s depressive symptoms, but external factors such as potential changes in instability may have simultaneously exacerbated household serious needs or daily stressors or depressive symptoms and the study does not allow for this unpacking without a control group. Second, while all efforts were made to create independence between the humanitarian organization and the research study, participants may have over-reported or under-reported certain outcomes, particularly if they thought it could result in further cash assistance. Although not directly involved in quantitative data collection, this may have been exacerbated as IRC research and program teams were closely involved in the collection of data due to security and logistical constraints of implementing a study in this setting. Qualitative interviews may have been particularly vulnerable to social desirability bias when two to three people (i.e., interviewer, note-taker, and, at times, a translator) were conducting the interview with a participant. Finally, the follow-up period encompasses the period immediately after the cash was likely to be spent; longer term follow up is needed to measure any sustained potential influences of cash.

Despite these limitations of inferring causality, this study offers the first rigorously conducted mixed methods evaluation of cash assistance programming inside Syria. Short-term emergency cash assistance programming yielded significant improvements in food security and assisted women and their families to meet their basic needs in the aftermath of pervasive traumatic experiences and challenges from the civil conflict and ISIS occupation. However, before and after this form of cash assistance was implemented, no meaningful changes in the perceived levels of serious needs or daily stressors amongst households were observed, but small increases in depressive symptoms for women were reported during this time period. Potential complementary programming models should be explored, including light-touch mental health programming models or referrals or integration of longer-term livelihoods programming for women as part of a broader exit strategy of cash assistance programming. Based on findings of this study, the IRC cash assistance teams in this setting have already worked to extend the length of cash assistance and develop referral systems and linkages to livelihoods programming. Further research is needed to determine appropriate targeting, length, and dosage of cash, alongside any potential complementary programming to yield these secondary positive changes to a cash assistance program focused on meeting population’s basic needs while not inadvertently delaying or decreasing reach of life-saving cash assistance programming in emergencies.

## Supporting information

S1 Table Demographic characteristics of qualitative sample (N = 40), previously published in Blackwell, A, et al. (2019) Women’s status and qualitative perceptions of a cash assistance programme in Raqqa Governorate, Syria. *Gender & Development 27(2)*: *253–71*.
(DOCX)

S2 Table Descriptive statistics of past-month food insecurity (HFIAS scale) at baseline (N = 512) and endline (N = 456). (DOCX)

S3 Table Descriptive statistics of adapted perceived needs and household stress (HESPER scale) at baseline (N = 512) and endline (N = 456). (DOCX)

S4 Table Descriptive statistics of past-two weeks depressive symptoms (PHQ-9 scale) at baseline (N = 512) and endline (N = 456). (DOCX)
