# Supplementary material for: Cash assistance programming and changes over time in ability to meet basic needs, food insecurity and depressive symptoms in Raqqa Governorate, Syria: Evidence from a mixed methods, pre-posttest
Source: PLoS One. 2020 May 7;15(5):e0232588. doi: 10.1371/journal.pone.0232588 (PMC7205216; doi:10.1371/journal.pone.0232588)
Supplement: S1 Table — Gender & Development 27(2): 253–71. (DOCX) [file pone.0232588.s001.docx]

Annex Table 1. Demographic characteristics of qualitative sample (N=40), **previously published in Blackwell, A, et al. (2019) Women’s status and qualitative perceptions of a cash assistance programme in Raqqa Governorate, Syria*. Gender & Development 27(2): 253-71.***

| Demographic Characteristic | N | %) |
| --- | --- | --- |
| Age  18-25  26-35  35+ | 7  15  18 | 18  38  45 |
| Relationship to head of household  Self  Wife  Daughter  Sister  Mother | 15  18  4  1  2 | 38  45  10  3  5 |
| Marital Status  Married  Divorced  Widowed | 24  5  11 | 60  13  28 |
| Displacement Status  Ever displaced  Currently displaced | 36  13 | 90  33 |
| Ever attended school  Yes  No | 27  13 | 68  33 |
