# Supplementary material for: Cash assistance programming and changes over time in ability to meet basic needs, food insecurity and depressive symptoms in Raqqa Governorate, Syria: Evidence from a mixed methods, pre-posttest
Source: PLoS One. 2020 May 7;15(5):e0232588. doi: 10.1371/journal.pone.0232588 (PMC7205216; doi:10.1371/journal.pone.0232588)
Supplement: S2 Table — (DOCX) [file pone.0232588.s002.docx]

Annex Table 2. Descriptive statistics of past-month food insecurity (HFIAS scale) at baseline (N=512) and endline (N=456).

| Food insecurity item  *In the past four weeks,* | Baseline  % (N) | Endline  % (N) |
| --- | --- | --- |
| Did you worry that your household would not have enough food | 81.5% (417) | 70.0% (319) |
| Were you or any household member not able to eat the kinds of foods you preferred because of a lack of resources | 92.8% (476) | 86.2% (393) |
| Did you or any household member have to eat a limited variety of foods due to a lack of resources | 91.0% (466) | 82.2% (375) |
| Did you or any household member have to eat some foods that you really did not want to eat because of a lack of resources to obtain other types of food | 89.8% (460) | 85.3% (389) |
| Did you or any household member have to eat a smaller meal than you felt you needed because there was not enough food | 84.0% (430) | 76.1% (347) |
| Did you or any household member have to eat fewer meals in a day because there was not enough food | 78.5% (402) | 69.1% (315) |
| Was there ever no food to eat of any kind in your household because of lack of resources to get food | 67.4% (345) | 53.3% (243) |
| Did you or any household member go to sleep at night hungry because there was not enough food | 62.1% (318) | 48.9% (223) |
| Did you or any household member go a whole day and night without eating anything because there was not enough food | 28.7% (147) | 12.5% (57) |
| *Mean Numbers of Items Agreed With (SD)* | *6.8 (2.2)* | *5.8 (2.4)* |
