# Supplementary material for: Cash assistance programming and changes over time in ability to meet basic needs, food insecurity and depressive symptoms in Raqqa Governorate, Syria: Evidence from a mixed methods, pre-posttest
Source: PLoS One. 2020 May 7;15(5):e0232588. doi: 10.1371/journal.pone.0232588 (PMC7205216; doi:10.1371/journal.pone.0232588)
Supplement: S3 Table — (DOCX) [file pone.0232588.s003.docx]

Annex Table 3. Descriptive statistics of adapted perceived needs and household stress (HESPER scale) at baseline (N=512) and endline (N=456).

| HESPER Item  *Do you have a serious problem because* | Baseline  % (N) | Endline  % (N) |
| --- | --- | --- |
| You do not have enough water that is safe for drinking or cooking? | 73.2% (375) | 74.3% (339) |
| You do not have enough food or because you are not able to eat. | 75.6% (387) | 72.4% (330) |
| You do not have a suitable place to live in. | 52.3% (268) | 61.8% (282) |
| You do not have easy and safe access to a clean toilet. | 44.7% (229) | 49.1% (224) |
| In your situation it is difficult to keep clean? For example, because you do not have enough soap, sanitary materials, water or a suitable place to wash. | 74.4% (381) | 73.9% (337) |
| You do not have enough, or good enough, clothes, shoes, bedding or blankets | 77.5% (397) | 64.0% (292) |
| You do not have enough income, money, or resources to live? | 91.4% (468) | 91.5% (417) |
| You have a problem with your physical health, like a physical illness, injury, or disability. | 59.4% (304) | 64.3% (293) |
| You are not able to get adequate health care for yourself such as treatment or medicines, health care during | 70.1% (359) | 72.6% (331) |
| Your children are not in school or are not getting good enough education. | 30.7% (157) | 41.5% (189) |
| You are very distressed. For example, very upset, sad, worried, scared, or angry. | 91.0% (466) | 89.5% (408) |
| You or your family are not safe or protected where you live now, for example, because of conflict, violence or crime in your community, city, or village. | 39.1% (200) | 29.8% (136) |
| It is difficult to care for family members who live with you? For example, young children in your family or family members who are elderly, physically or mentally ill, or disabled. | 48.8% (250) | 48.5% (221) |
| You are not getting enough support from people in your community? For example, emotional support or practical help. | 38.9% (199) | 54.6% (249) |
| You have been displaced from your home country, city, or village. | 59.0% (302) | 72.2% (329) |
| You do not have enough information? For example, about the aid that is available or what is happening in your home community. | 66.6% (341) | 61.0% (278) |
| Inadequate aid? For example, you do not have fair access to aid that is available. | 71.7% (367) | 57.5% (262) |
| You do not feel respected or you feel humiliated? | 33.6% (172) | 23.5% (107) |
| You are not able to move between places. | 56.8% (291) | 59.4% (271) |
| You have too much free time in the day. | 52.7% (270) | 50.4% (230) |
| *Mean HESPER Items Agreed With (SD)* | *12.08 (3.32)* | *12.11 (3.87)* |
