# Supplementary material for: Cash assistance programming and changes over time in ability to meet basic needs, food insecurity and depressive symptoms in Raqqa Governorate, Syria: Evidence from a mixed methods, pre-posttest
Source: PLoS One. 2020 May 7;15(5):e0232588. doi: 10.1371/journal.pone.0232588 (PMC7205216; doi:10.1371/journal.pone.0232588)
Supplement: S4 Table — (DOCX) [file pone.0232588.s004.docx]

Annex Table 4. Descriptive statistics of past-two weeks depressive symptoms (PHQ-9 scale) at baseline (N=512) and endline (N=456).

| PHQ-9 Scale  Over the last 2 weeks, how often have you been bothered by any of the following problems? | Baseline  % (N) | | | | Endline  % (N) | | | |
| --- | --- | --- | --- | --- | --- | --- | --- | --- |
|  | Not at All | Several Days | More than Half the Days | Nearly Every Day | Not at All | Several Days | More than Half the Days | Nearly Every Day |
| Little interest or pleasure in doing things | 20.7% (106) | 43.8% (224) | 15.4% (79) | 20.1% (103) | 15.6% (71) | 41.7% (190) | 19.7% (90) | 23.0% (105) |
| Feeling down, depressed, or hopeless | 6.8% (35) | 42.2% (216) | 19.5% (100) | 31.5% (161) | 8.8% (40) | 35.1% (160) | 22.8% (104) | 33.3% (152) |
| Trouble falling or staying asleep, or sleeping too much | 30.9% (158) | 32.6% (167) | 12.1% (62) | 24.4% (125) | 29.8% (136) | 32.5% (148) | 19.5% (89) | 18.2% (83) |
| Feeling tired or having little energy | 6.5% (33) | 46.5% (238) | 19.7% (101) | 27.3% (140) | 7.9% (36) | 33.2% (151) | 22.6% (103) | 36.4% (166) |
| Poor appetite or overeating | 25.4% (130) | 35.4% (181) | 17.8% (91) | 21.5% (110) | 29.2% (133) | 32.9% (150) | 19.4% (88) | 18.3% (85) |
| Feeling bad about yourself - or that you are a failure or have let yourself or your family down | 44.1% (226) | 31.8% (163) | 10.2% (52) | 13.9% (71) | 40.8% (186) | 26.1% (119) | 12.1% (55) | 21.1% (96) |
| Trouble concentrating on things, such as reading the newspaper or watching television | 26.0% (133) | 43.0% (220) | 15.4% (79) | 15.6% (80) | 15.56 (71) | 46.3% (211) | 19.3% (88) | 18.9% (86) |
| Moving or speaking so slowly that other people could have noticed? Or the opposite — being so fidgety or restless that you have been moving around a lot more than usual | 26.2% (134) | 47.3% (242) | 17.0% (87) | 9.6% (49) | 18.4% (84) | 51.3% (234) | 16.2% (74) | 14.0% (64) |
| Thoughts that you would be better off dead or of hurting yourself in some way | 73.6% (377) | 17.6% (90) | 5.1% (26) | 3.7% (19) | 73.0% (333) | 18.2% (83) | 4.0% (18) | 4.8% (22) |
| *Mean Depressive Symptoms (SD)* | 11.07 (5.02) | | | | 11.95 (5.08) | | | |
